# Outcomes of Using IVC Filters in Patients with Malignancy at an Academic Medical Center

**DOI:** 10.1055/s-0039-1688569

**Published:** 2019-04-29

**Authors:** Prashant Raghavendran, Ming Y. Lim

**Affiliations:** 1 Division of General Internal Medicine, Department of Medicine, Medical University of South Carolina, Charleston, South Carolina, United States; 2 Division of Hematology/Oncology, Department of Medicine, Medical University of South Carolina, Charleston, South Carolina, United States

**Keywords:** anticoagulation therapy, cancer, thrombosis, survival, outcomes

## Abstract

Systemic anticoagulation is regarded as optimal treatment and prophylaxis of venous thromboembolism (VTE). In malignancy, bleeding risk is increased while the patients remain hypercoagulable, making anticoagulation management troublesome. Inferior vena cava (IVC) filters have emerged as an option in the management of VTE, especially when anticoagulant agents are contraindicated. There is limited data on the overall outcomes of patients with malignancy and IVC filter placement. This descriptive study identifies individuals with filters placed and reviews outcomes to guide appropriate care of patients with malignancy and VTE. We performed a retrospective chart review of 115 patients with malignancy who had a filter placed between July 2014 and December 2016. Eighty-seven patients were tracked until December 2017 for significant events (VTE and/or death). In total, 61% ( *n* = 70) had metastatic solid tumor malignancy and 77% ( *n* = 88) were receiving anticoagulation therapy prior to IVC filter placement. Fifty-three percent ( *n* = 61) had bleeding events and 25% ( *n* = 29) had thrombocytopenia. Patients with isolated solid tumors receiving frequent surgery were also common recipients of filters. Sixty-six percent (57/87) of patients had a significant event; 85% of them were anticoagulated. Eighty-two percent of events occurred within 6 months of filter placement, with death occurring on average within 5 months of placement. Overall, use of IVC filters was more common in cancer patients who developed bleeding complications on anticoagulation and with metastatic malignancy. However, in patients with metastatic or hematologic disease, filter placement did not prevent all-cause mortality. Individualized risk–benefit consideration is needed before IVC filters are placed.

## Introduction

Anticoagulation is the optimal treatment for venous thromboembolism (VTE) and for prophylaxis of VTE in high-risk patients. 1 However, the use of anticoagulation can be challenging in patients with cancer. Malignancy is a circumstance in which risk of bleeding is increased while patients remain in a hypercoagulable state. 2 VTE occurs in 15% of patients with cancer, with malignancy conferring a sevenfold increase in risk of clotting compared with healthy individuals. 3 Conversely, hemorrhage occurs in up to 10% of patients with malignancy, and this is often precipitated by use of anticoagulant therapy. 4 Overall, thromboembolism and hemorrhage account for 18% of mortality in these individuals. 3

Furthermore, patients with previous VTE events may experience end-organ damage such as cardiac strain, pulmonary compromise, and perfusion defects to other organs that raises concern for future pathology. In this context, providers place inferior vena cava (IVC) filters in patients to prevent cardiopulmonary thrombotic events, especially after a bleeding event occurred on anticoagulant therapy. 5 IVC filters have emerged as an option to prevent pulmonary embolus (PE) in patients with existing deep venous thrombosis (DVT) or as prophylaxis in patients for whom anticoagulation is contraindicated. 6 There is limited data in exploring the types of patients with cancer that receive these devices and overall outcomes after placement. Continued compilation of this information can help guide the care of these patients with complex coagulopathic states.

The aim of this study is to provide insight into patients with malignancy that received IVC filters and to review the outcomes of patients after the procedure for pathologic events and/or mortality to aid with future clinical decision-making.

## Methods

A retrospective chart review was performed on patients with malignancy who had an IVC filter placed between July 1, 2014 and December 31, 2016. Demographic data were collected on patients, including ethnicity, gender, and age. Individuals were divided into three categories of malignancy: isolated solid tumor, metastatic solid tumor, and hematologic malignancies. The following data were collected: history of VTE, including DVT, PE, or other venous thrombus; previous anticoagulation therapy; bleeding events (defined as intracranial, intraperitoneal, genitourinary, hematoma, etc.); platelet count on the day prior to the procedure; and type of IVC filter (retrievable vs. nonretrievable) placed.

Patients were observed until December 2017 to provide at least a 1-year postintervention window for all patients. Patients were tracked for VTE events (initial and recurrent) and death, which were the primary significant events. Time to the primary significant event from IVC filter placement was calculated in weeks. Time to demise for all patients was also recorded. The following data were also collected: cause of death (if available); whether the patient was on concurrent anticoagulation therapy and IVC filter placement; and time to IVC filter removable (if applicable).

## Results

This was a descriptive retrospective cohort study. A total of 115 patients with malignancy had an IVC filter placed during the study period ( Table 1 ). There was near equal distribution between African-Americans ( *n* = 59) and Caucasians ( *n* = 56). Forty-seven males and 68 females were present in the initial cohort. In total, 61% ( *n* = 70) were noted to have metastatic solid tumor malignancies, 27% ( *n* = 31) isolated solid tumor malignancies, and 12% ( *n* = 14) hematologic malignancies. Of the 115 patients, 97% ( *n* = 111) had a preceding VTE, and 76% ( *n* = 87) were anticoagulated prior to IVC filter placement. Enoxaparin was used most commonly ( *n* = 39, 44%); warfarin ( *n* = 17, 19%), direct oral anticoagulants (rivaroxaban and apixaban) ( *n* = 14, 16%), and inpatient heparin infusions ( *n* = 15, 17%) also were used frequently ( Table 2 ). Fifty-three percent ( *n* = 61) of the cohort had a bleeding event; of which 79% ( *n* = 48) were on anticoagulation therapy. In addition, 19% ( *n* = 22) of patients were scheduled to undergo several tumor debulking surgeries in the months following IVC filter placement. Twenty-five percent ( *n* = 29) had thrombocytopenia with platelet count <150,000/μL with four patients having moderate thrombocytopenia with platelet count <50,000/μL. Patients with hematologic malignancies were more likely to have lower platelet counts than those with other types of malignancy. Three patients had a documented fall history. Twenty-eight percent ( *n* = 32) of patients were lost to follow-up in the observation period; 4 of these patients had a significant event occur but were then lost to follow-up, thus limiting the cohort to 87 patients who were followed until December 2017.

**Table 1:** Demographic data for patients in the initial cohort

	Metastatic solid tumor patients	Isolated solid tumor patients	Hematologic malignancy patients	Total
Number of patients	70 (61%)	31 (27%)	14 (12%)	115
African-American	40 (57%)	11 (35%)	8 (57%)	59 (51%)
Caucasian	30 (43%)	20 (65%)	6 (43%)	56 (49%)
				Total
Age: <50 y	14 (20%)	2 (6%)	4 (29%)	20 (17%)
Age: 50–70 y	40 (57%)	22 (71%)	7 (50%)	69 (60%)
Age: >70 y	16 (23%)	7 (23%)	3 (21%)	26 (23%)
				Total
Male	28 (40%)	10 (32%)	9 (64%)	47 (41%)
Female	42 (60%)	21 (68%)	5 (36%)	68 (59%)
				Total
Previous VTE	67 (96%)	30 (97%)	14 (100%)	111 (97%)
Previous DVT	31	13	9	53
Previous PE	19	7	2	28
Multiple previous thrombi	17	10	3	30
				Total
Previously anticoagulated	53 (76%)	25 (81%)	10 (71%)	88 (77%)
Not previously anticoagulated	17 (24%)	6 (19%)	4 (29%)	27 (23%)
Previous bleed	40 (57%)	14 (45%)	7 (50%)	61 (53%)
Previous bleed on anticoagulation	31 (78%)	11 (79%)	6 (86%)	48
				Overall average
Average platelet count prior to filter placement (per μL)	259,000	243,000	100,000	235,000
				Total
Thrombocytopenic patients (<150,000/μL)	13 (19%)	4 (13%)	12 (86%)	29 (25%)
Severely thrombocytopenic patients (<50,000/μL)	1 (1%)	0	3 (21%)	4 (3.5%)
Documented fall history	2 (3%)	0	1 (7%)	3 (3%)

Abbreviations: DVT, deep venous thrombosis; PE, pulmonary embolus; VTE, venous thromboembolism.

**Table 2:** Description of anticoagulation therapies of patients prior to IVC filter placement

Anticoagulation prior to IVC filter placement	Enoxaparin	Rivaroxaban	Apixaban	Bivalirudin	Warfarin	Unfractionated heparin infusion	Subcutaneous unfractionated heparin
Metastatic solid tumor patients	24	4	1	0	13	11	1
Isolated solid tumor patients	11	6	1	0	2	2	0
Hematologic malignancy patients	4	2	0	1	2	2	0
Total	39 (44%)	12 (14%)	2 (2%)	1 (1%)	17 (19%)	15 (17%)	1 (1%)

Abbreviation: IVC, inferior vena cava.

**Fig. 1:**
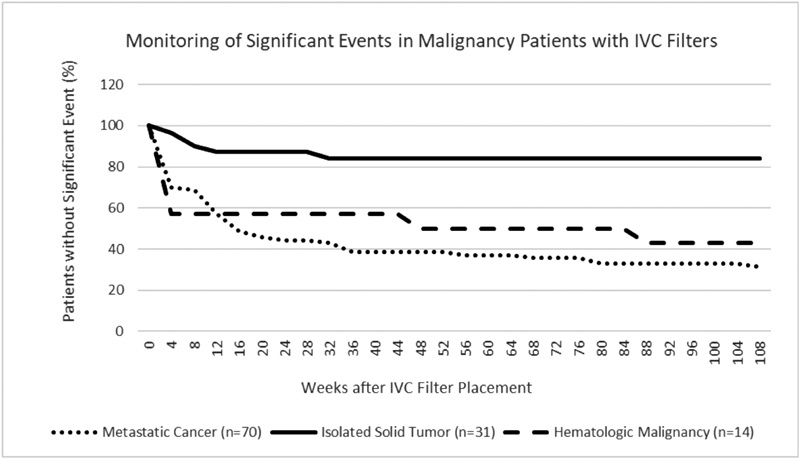
Plot representing incremental significant event-free survival of patients after IVC filter placement, apportioned by category of malignancy. IVC, inferior vena cava.

**Fig. 2:**
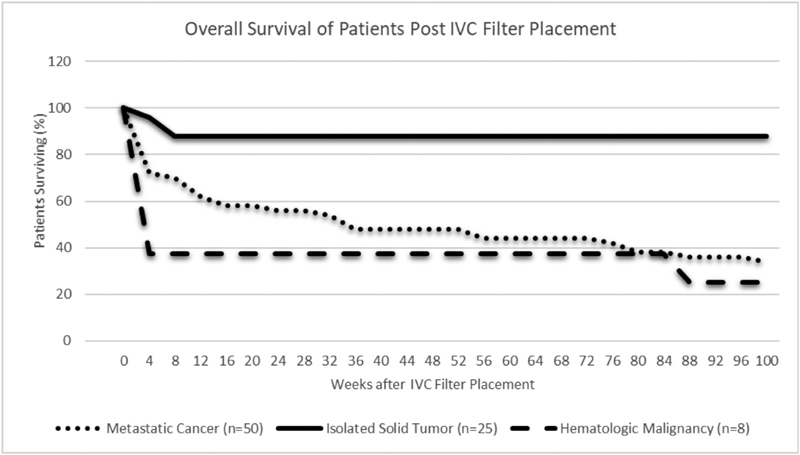
Plot representing incremental survival after all-cause mortality during the observation period, apportioned by category of malignancy.

Median time to follow-up for patients was 12 weeks after filter placement (range: 1–52 weeks). Sixty-six percent of the patients that remained in the cohort (57/87) had a significant event in the observation period. This occurred in 81% of patients with metastatic malignancy (44/54) and 88% with hematologic malignancy (7/8), but only 20% of those with isolated solid tumor (5/25). Most significant events were deaths ( *n* = 43, 75% of events); 8 additional patients died in the observation period after developing thrombus, totaling to 52 deaths (45% of all patients). Fifteen percent of patients had VTE after filter placement ( *n* = 13/87), with 10% of patients ( *n* = 8/87) still having pulmonary embolism after the procedure. Despite 85% ( *n* = 74/87) of patients being on anticoagulation therapy, 69% ( *n* = 9) of VTEs occurred while on anticoagulation therapy. The mean and median time to a significant event from IVC filter placement were 14 and 4 weeks, respectively (range: 0–108 weeks). Most earlier events occurred in the metastatic solid tumor malignancy group ( Table 3 , Figs. 1 and 2 ). Complications of the malignancy itself were cited as the cause of death in 75% of the 52 patients who died during observation ( *n* = 39), with VTE alone being responsible for only 6% ( *n* = 3) of mortality; bleed and unknown factors were listed as causes for the other 19%. IVC filters placed were nearly all retrievable models ( *n* = 110, 96%), but only 14% ( *n* = 16) were removed, with a majority of those removed ( *n* = 11, 69%) occurring in the isolated solid tumor malignancy group. The mean duration to removal from initial placement was 29 weeks (range: 0–96 weeks), with the median removal time being 17 weeks.

**Table 3:** Outcomes after IVC filter placement

	Metastatic solid tumor patients	Isolated solid tumor malignancy patients	Hematologic malignancy patients	Total
Total initial patients in the cohort	70	31	14	115
Patients observed through full period	50	25	8	83
Patients with significant event before loss to follow-up	4	0	0	4
Denominator for outcome calculations	54	25	8	87
Patients lost to follow-up	20	6	6	28
				Total
Anticoagulated after IVC filter	42 (60%)	25 (81%)	7 (50%)	74 (64%)
				Total
Significant event	44 (81%)	5 (20%)	7 (88%)	57 (66%)
Pulmonary embolism	6 (12%)	0	2 (25%)	8 (9%)
On anticoagulation	4 (67%)	0	1 (50%)	5 (63%)
Deep venous thrombus	3 (6%)	2 (8%)	0	5 (6%)
On anticoagulation	3 (100%)	1 (50%)	0	4 (80%)
Death	35 (70%)	3 (12%)	5 (62.5%)	43 (49%)
Death after thrombus	8 (16%)	0	0	8 (9%)
No recorded events	6 (19%)	20 (80%)	1 (12%)	27 (34%)
Causes of death				
Malignancy	39 (75%)			
Multifactorial	7 (13%)			
VTE	3 (6%)			
Bleed	3 (6%)			
Total deaths	52			

Abbreviations: IVC, inferior vena cava; VTE, venous thromboembolism.

## Discussion

Our study demonstrates that death from either VTE or other complications on average occurs at a high rate in patients with malignancy and IVC filter placement. Given that pathologic episodes—especially death—occur on average within 3 to 4 months of device placement, existing comorbidities in malignancy still confer significant risk for thrombus and hence, mortality despite blockade devices. Especially in the case of metastatic disease, mortality risk is already elevated, so comorbidities of malignancy alone contribute to mortality even without thrombus. Our cohort showed evidence of thrombus even with concurrent chemical anticoagulation, echoing existent literature that recurrent VTE occurs in 20% of malignancy patients on medication. 7 From our data, with isolated solid tumor malignancy, adverse outcomes seem to be avoided more often; however, in the setting of metastatic or hematologic malignancy where disease is more widespread, risk and outcomes are individualized despite device placement. Along these lines, practitioners are likely, especially when there is surgical curative intent, to place filters and remove them after the window of malignancy risk has passed. 8 Otherwise, previous clot increases predilection for future thrombus and when bleed is easily precipitated by anticoagulation, an anatomical blockade in the form of a vena cava filter is seen as the only way to prevent lethal VTE in the very fine line between thrombosis and coagulopathy. 9

The PREPIC study showed that patients with IVC filter in place and receiving anticoagulation therapy had an increased risk of DVT but lower risk of PE, likely leading to an increased consideration for the devices; however, long-term mortality and morbidity was similar in patients with filters and without, thus questioning the overall benefit. 10 More specifically with malignancy, literature indicates persistent pathological burden even with device placement. In one study, nearly half of the patients who had stage IV malignancy and an IVC filter present died within 6 weeks of placement, so risk reduction could not be measured. 11 In another study examining 206 patients with malignancy and VTE, patients with IVC filter alone as compared with those with anticoagulation had a nearly twofold increased risk of all-cause mortality. 12 Given this, practitioners who decide to use IVC filters for therapy must understand that it is a temporary option, with planned removal of the IVC filter once no longer clinically indicated. It has unfortunately been noted, however, that when filters are placed, there are no clear monitoring processes in place to determine the utility of continued deployment versus retrieval of the device, making appropriate follow-up and removal difficult. 9 Ultimately, the use of IVC filters for anticoagulation therapy in cancer patients is not generalizable and requires risk–benefit analysis for each individual patient.

Further data exist to indicate situations of potential benefit from IVC filters and the importance of individualizing decisions to place them. One study evaluated the outcomes in 50 patients with DVT and malignancy who had IVC filters placed; most cases had a previous history of contraindication to anticoagulation or bleed on anticoagulation therapy, with a sizeable minority with noted PE while on anticoagulation or thrombus that caused significant cardiopulmonary compromise. When filters were placed, there were minimal thrombotic events such as PE or IVC thrombus noted (with most patients also on anticoagulation), but 40% of the patients in the cohort had passed away within the observation period from metastatic malignancy. 13 Therefore, the preventative efficacy of filter placement alone could not be elucidated due to the overall high mortality rate of these patients with malignancy and contribution of systemic anticoagulation. Another study evaluated a cohort of 55 patients with late-stage malignancy and history of VTE that had an IVC filter placed. In this group, 24% patients survived during the 1-year observation period and 24% of the patients died. Those with more severe disease were predisposed to having decreased ability to ambulate and maneuver and thus were more likely to die from the effect of VTE or other causes than those with less invasive disease. 14 Based on this information, the overall functional status should be a consideration for individual prognostication and can help precipitate a decision toward placing a filter.

However, current data suggest that when appropriate, anticoagulation is noninferior to IVC filters in preventing life-threatening thrombosis. A retrospective study of 166 patients with malignancy was performed to evaluate mortality outcomes for 1 year after they were divided into anticoagulation and IVC filter cohorts. At conclusion of observation, the filter group had a 35% survival rate while the anticoagulation group had a 38% survival rate. 15 Since survival rates were similar between the two groups, anticoagulation should still be considered optimal for the hypercoagulable state of malignancy. Furthermore, a more recent retrospective meta-analysis of over 35,000 patients showed a nearly threefold increase in relative risk of adverse thrombotic event or mortality with IVC filter placement versus chemical anticoagulation in malignancy patients. 16 Analysis has revealed that IVC filters should not be inserted if chemical anticoagulation is viable. If filter placement is considered, a thorough risk–benefit discussion should occur with each patient. Survival is overall poor in all individuals however, indicating that the best policy would be to advocate for the line of therapy which best mirrors the patient's wishes.

## Study Limitations

This is a small study with only 115 patients, which limits the power and significance of the data. Because this analysis was retrospective, data collection depended on patient correspondence with health care providers and accurate documentation in the medical records. Given the number of patients lost to follow-up in the observation period, an accurate eventual outcome was uncertain in nearly 25% of the cohort as well, further limiting the data.

## Conclusion

It is often difficult to glean the contribution of VTE to overall morbidity and mortality in patients with multiple other comorbidities, including malignancy. It is often a situation of heightened acuity and exhaustion of alternative therapies due to treatment failure or bleeding that prompts use of IVC filters. Further considerations include persistent thrombocytopenia and recurrent surgery requiring temporary cessation of anticoagulation therapy. However, given overall prognosis of patients with malignancy, a very thorough, multidisciplinary conversation should occur to evaluate a patient's functional status, therapeutic options, and the current state of oncologic disease before placement of such devices. Existing data confirm that mortality rates, either from VTE or other causes, remain high after placement of IVC filters in patients with aggressive malignancy. Our hope is that this information adds to a broader discussion of situations that would prompt consideration of this device. The goal would be for other medical centers to analyze similar patient populations and add to the significance of existing data. A more generalized understanding of therapy options in the delicate balance between clotting and bleeding in malignancy is vital in ensuring safer patient care.
